# Serious mental illness and negative substance use consequences among adults on probation

**DOI:** 10.1186/s40352-018-0064-7

**Published:** 2018-03-22

**Authors:** Matthew E. Rossheim, Melvin D. Livingston, Jennifer A. Lerch, Faye S. Taxman, Scott T. Walters

**Affiliations:** 1 Department of Global and Community Health, George Mason University, 4400 University Drive, MS5B7, Robinson Hall B, Fairfax, VA 22030-4444 USA; 2 Department of Biostatistics and Epidemiology, University of North Texas Health Science Center, Fort Worth, TX USA; 3 Department of Criminology, Law & Society, Center for Advancing Correctional Excellence!, George Mason University, Fairfax, VA USA; 4 Department of Health Behavior and Health Systems, University of North Texas Health Science Center, Fort Worth, TX USA

**Keywords:** Bipolar disorder, Major depressive disorder, Schizophrenia, Substance use

## Abstract

**Background:**

Adults on probation are at greater risk of both using substances and having a mental disorder compared to the general population. Several theories explain the relationship between substance use and poor mental health. However, the interaction between substance use, mental health, and substance-related consequences is not well understood. A better understanding of this relationship may help treatment programs become more responsive to people with serious mental illness (SMI).

**Method:**

The current study used interview data from 313 adults on probation who reported recent substance use. We examined associations between SMI risk, substance use, and substance use consequences.

**Results:**

A substantial proportion of the sample (37.5%) screened at risk of having a SMI. Adjusting for type and amount of substance use, those who screened at risk of having a SMI reported more negative substance use consequences. Significant interaction effects were observed between use of alcohol or opiates and SMI risk. Alcohol use was associated with more negative substance use consequences among those at risk of SMI, while opiate use was associated with more consequences among those not at risk.

**Conclusions:**

Programs are sorely needed to identify and treat adults with comorbid substance use and mental health symptoms, particularly for adults in the justice system. Clinicians should carefully consider how mental health may interact with substance use to exacerbate consequences.

## Background

Serious Mental Illness (SMI) most often refers to a diagnosis of major depression, schizophrenia, or bipolar disorder (Center for Behavioral Health Statistics Quality, 2015; Kessler et al., 2003; Mueser et al., 1998). According to the *National Survey on Drug Use and Health*, in 2014, an estimated one in four U.S. adults with SMI also had a substance use disorder (SUD; Center for Behavioral Health Statistics and Quality, 2015). Given that 8.5% of U.S. adults had a SUD, the rate of SUDs among those with SMI is roughly three times that of the general adult population (SAMHSA, 2014a).

There are a number of explanations for the co-existence of substance use and poor mental health. First, substance use and mental health disorders may co-occur as a result of similar social-, environmental-, behavioral-, and trait-level factors (Chilcoat & Breslau, 1998; Mueser et al., 1998). For example, conduct disorder and antisocial personality disorder are strongly associated with the development of both substance use and SMI; however, these associations may be due in part to other common social or cognitive factors (Mueser et al., 1998). Second, people with mental illness may “self-medicate” by using substances to alleviate the consequences and/or social issues caused by their mental health symptoms (Chilcoat & Breslau, 1998; Harris & Edlund, 2005; Mueser et al., 1998). Third, through pharmacological reactions to use or withdrawal, substance use can cause psychiatric symptoms that, when clustered and sustained over time, develop into induced psychiatric syndromes (American Psychiatric Association, 2013; Shivani et al., 2002). Fourth, according to the supersensitivity hypothesis, some people with SMI may be genetically predisposed to experience heightened psychobiological responses to substances, potentially including increased dopamine transmission, which promotes repeated use (Drake & Mueser, 2002; Mueser et al., 1998). These theories are not mutually exclusive; each may provide some insight on the development of these co-occurring conditions.

Historically, much of the substance abuse treatment field promoted abstinence-based policies. However, a recent study of 432 addiction and mental health professionals in the U.S. found that a large proportion viewed non-abstinence (i.e., limited or moderate use) goals as acceptable for some clients, especially as an intermediary goal for those diagnosed with alcohol (44%) or cannabis (43%) abuse (Rosenberg & Davis, 2014). Nearly one in ten providers also viewed moderation as an acceptable long-term goal for people with diagnosed dependence for amphetamines (9%), heroin (9%), cocaine (8%), and MDMA/ecstasy (9%) (Rosenberg & Davis, 2014). These non-abstinence policies often recommend safer, less frequent use of substances, for instance using less of a drug or using a safer route of administration (Bigg, 2001; Lenton & Single, 1998).

Although harm reduction practices can benefit some people, the biological vulnerabilities and heightened psychobiological responses to substance use among SMI clients tempers this conclusion (Mueser et al., 1998). In fact, a level of substance use that generally does not result in consequences for most people may result in consequences for those with SMI (Drake & Mueser, 2002; Mueser et al., 1998). Thus, in order to provide the best advice, practitioners need information regarding the type and amount of substance use that result in consequences for different kinds of people.

Drake and Brunette (1998) reviewed more than 100 cross sectional and prospective studies examining consequences associated with having a SUD among people with SMI. They concluded that, among people with SMI, having a SUD is associated with greater risk for consequences across several dimensions of life including: mental and physical health, functional status, problems with family, residential stability, and disruptive behavior (Drake & Brunette, 1998). However, since SUDs are in part diagnosed by assessing problematic patterns of use (American Psychiatric Association, 2013) it is unclear whether substance use increases consequences among those with SMI or whether meeting criteria for a SUD is simply an artifact of experiencing more consequences. Limited research has examined associations between SMI and consequences experienced among people who use substances (Gonzalez et al., 2007), who tend to experience more life consequences than people who do not use substances. More research is needed to compare, among people who use substances, interactions between substance use, SMI, and substance-related consequences.

In the current study, we examined the interaction between screening at risk of SMI, substance use, and substance use consequences experienced by a sample of adults who were newly placed on probation. Adult probationers are a group of interest for several reasons. First, probation is the largest segment of the U.S. justice system, encompassing nearly 4 million adults (Kaeble et al., 2015). Second, adult probationers are much more likely to use illicit drugs (31.4%) compared to the general adult population (9.0%) (SAMHSA, 2014b). Finally, people on probation are also at greater risk of having a mental health disorder compared to the general population (Feucht & Gfroerer, 2011; SAMHSA, 2014a).

Self-reported negative consequences of substance use reflect both the magnitude of consequences experienced and self-recognition that substance use is problematic. According to widely-used health behavior theories, recognizing that one’s own substance use is problematic is an important first step towards behavior change (Prochaska et al., 1992). Further, people who report more negative consequences from substance use (e.g., social and health consequences) are more likely to seek treatment (Hajema et al., 1999; Kaskutas et al., 1997; Oser et al., 2010; Saunders et al., 2006; Weisner et al., 2002). The current study provides insight on the extent to which people who use different substances and people who screen at risk of SMI versus those who do not attribute unfavorable experiences to their substance use, which likely influences their motivation for seeking treatment.

The current study offers a number of strengths over existing research. First, the majority of relevant research to date has focused on substance abuse/use disorders (Drake & Brunette, 1998), which (1) may be a marker of consequences experienced, (2) may be underdiagnosed, and (3) overlooks moderate use of substances. It may be problematic to overlook moderate substance use because even moderate use may cause consequences for individuals with SMI. Second, the comparison group in the current study were adults who used substances but did not screen at risk of SMI. In most previous research, the comparison groups had SMI but did not meet criteria for SUDs. Thus, findings from previous studies may reflect differences between adults who used substances and those who did not, rather than the effect of SMI among substance users (Drake & Brunette, 1998). Finally, rather than focusing on adults who used just one drug type, this study simultaneously considers the combination of substances used, giving it greater applicability to the context of the justice system, in which people often use two or more substances recreationally.

## Methods

### Study sample

The sample included 313 adults in Baltimore City, MD and Dallas, TX who were participating in a larger clinical trial (Taxman et al., 2015). Participants were recruited during the probation intake/orientation process using convenience and snowball sampling. Eligible participants were at least 18 years old, spoke English, were newly on probation (within 30 days of their court date), and reported heavy alcohol use (5/4 drinks for men/women) or any illicit drug use during the past 90 days. A total of 2307 adults were screened for eligibility. Of those, 1599 were ineligible for study participation, primarily because: they did not meet substance use criteria, their court date for probation sentencing was more than 30 days ago, and/or they were not on probation. An additional 392 participants who were eligible did not complete the baseline interview, most often because researchers were unable to contact them, they did not appear for the interview, or they voluntarily withdrew from the study. An additional 3 participants who completed the survey declined to provide complete information necessary for data analyses. As a result, the final study sample included 313 participants (180 from Dallas and 133 from Baltimore).

### Measures

#### Independent variables

#### Substance use items

Past 90-day drug and alcohol use data were gathered using the timeline followback (TLFB) method (Sobell & Sobell, 1996). In previous studies, the TLFB method demonstrated strong reliability and validity when compared to other measures of substance use (Fals-Stewart et al., 2000), including among homeless people (Sacks et al., 2003) and psychiatric outpatients (Carey, 1997). Clients were asked how many days they had used each illicit substance over the past 90 days: marijuana, opiates, cocaine/crack, amphetamines, prescription pills, hallucinogens, inhalants, barbiturates, and other drugs. Alcohol use was measured as the number of standard drinks consumed on a given day (i.e., each 14 g of ethanol comprised one standard drink). Using these data, a variable for total number of binge drinking episodes was created. Binge drinking was defined as consuming 5 or more drinks on a single day for men or 4 or more drinks for women.

Dichotomous variables were created using these continuous substance use variables. This was done to better distinguish between meaningful cut-offs in substance use. For example, the difference between consuming 0 drinks and 1 drink is not the same as the difference between consuming 4 and 5 drinks. Use of each substance type was categorized based on frequency of use: non-use, moderate use, and heavy use. People who used each drug were divided into two groups of nearly equal size. *Moderate use* was defined as using a substance on a number of days below the median level (among all people who used the drug), whereas *heavy use* was defined as using a substance on a number of days above the median level. Use of a median split for heavy/moderate substance use is common in the research literature and was used for the current study because standard cutoffs for the general population may not be normative for people in the justice system (Field et al., 2011; Montgomery et al., 2012; Papachristou et al., 2012; Witbrodt et al., 2014). Heavy use over the last 90 days was defined as, at least: prescription pill use on 6 days, binge drinking on 7 days, amphetamine use on 8 days, crack/cocaine use on 9 days, opiate use on 27 days, and marijuana use on 34 days.

#### Risk of serious mental illness

The Co-occurring Disorders Screening Instrument for Severe Mental Disorders (CODSI-SMD) is a three item screener for any SMI (i.e., major depression, schizophrenia, and bipolar disorders) during a person’s lifetime. In previous research, the CODSI-SMD with a cut-off score of 2 has demonstrated greater accuracy in identifying people with any DSM-IV measured SMI than other commonly used instruments (Sacks et al., 2007b; Sacks et al., 2007a). This instrument and cut-off score were used to classify participants as *at risk* or *not at risk* of having SMI.

#### Risk taking

According to the concept of “problem behavior syndrome,” some people have an increased proclivity for risk-taking (Jessor et al., 1991; Moraleda-Barreno, 2018). As a result, individuals may be more apt to use substances and experience more negative life consequences. To account for this, items from the Texas Christian University Criminal Justice Client Evaluation of Self and Treatment (CJ CEST) risk taking sub-scale were included as a continuous variable in data analyses. In previous assessments, the CJ CEST demonstrated good reliability and validity among people in the justice system who use substances (Garner et al., 2007). Within the study sample, the risk taking sub-scale demonstrated adequate internal consistency reliability (α = 0.68), comparable to values observed with other samples (α = 0.71) (Garner et al., 2007).

#### Dependent variable

The 15-item Short Inventory of Problems (SIP) has been used in a variety of settings to assess negative substance-use consequences across multiple life domains (Kiluk et al., 2013). The SIP shows excellent internal consistency and is highly correlated with the longer Inventory of Drug Use Consequences (Bennett et al., 2009). For the current study, we created a modified, 18-item version of the SIP (adding 3 questions about legal consequences to the original scale) that asks about negative consequences resulting from either alcohol or drug use (i.e., physical, interpersonal, intrapersonal, risky behaviors, responsibilities, and legal). Prior to listing consequences, participants were read the following prompt, “Thinking about the last 3 months, how often have each of the following happened to you because of your drinking or drug use”. Response options (and their corresponding scores) were: “never” (0), “once or a few times” (1), “once or twice a week” (2), “daily or almost daily” (3). Responses for each item were summed to generate an overall score for negative substance use consequences. Thus, scores could range from 0 to 54.

#### Statistical analyses

First, descriptive statistics were used to describe the study sample. Next, bivariate linear models were used to estimate the crude association between each predictor and the negative consequences of substance use, and multivariable linear models were used to estimate the independent effect of each predictor controlling for the remaining variables. These analyses were important for two reasons. First, they allowed for the examination of associations between SMI risk and negative substance use consequences – adjusting for substance use – prior to testing interaction effects. Second, negative substance use consequences are likely substance-specific. Therefore, the adapted scale used in this study could be better at identifying consequences related to only some substances. These analyses helped gauge whether the negative substance use consequences variable would detect consequences experienced from the use of different substances. Finally, we assessed whether there were differential effects of each substance use by SMI risk using a series of models including an interaction term between SMI risk and each substance use measure. All continuous variables were standardized to aid interpretation of the results.

## Results

### Sample characteristics

The majority of the sample were men (66.1%), Black (68.1%), and non-Hispanic (79.9%). The average participant age was nearly 35 years. A substantial proportion of the sample screened at risk of serious mental illness (37.5%). Results from bivariate tests indicated no statistically significant differences between those who screened at risk and not at risk of SMI with regard to age or race (*p* > 0.05). However, those at risk of SMI were significantly more likely to be women and non-Hispanic, compared to those not at risk (*p* <  0.05).

Eligible participants reported at least 1 day of illicit drug use or heavy alcohol use during the past 90 days. Heavy drinking was common (65.5%), and a substantial proportion of the sample reported using marijuana (63.6%), crack/cocaine (31.0%), opiates (19.8%), prescription pills (14.7%), and amphetamines (8.0%). Use of hallucinogens (3.2%), inhalants (1.0%), barbiturates (0.3%), and other drugs (0.6%) were far rarer, and therefore not included in subsequent analyses.

Scores on the substance use consequences measure ranged from 0 to 50, with an average score of 16.1. This scale demonstrated very good internal consistency (α = 0.926). The alpha was highest when all items were included; removal of any item did not improve inter-item reliability.

### Main effects model

Bivariate models showed a significant association between SMI risk and substance use consequences (β = 0.76, 95% CI = 0.55, 0.97), as well as between substance use and substance use consequences (Table 1). Compared to non-opiate use (i.e., people who drank alcohol and/or used other illicit drug types), both heavy opiate (β = 1.32, 95% CI = 0.99, 1.65) and moderate opiate (β = 0.68, 95% CI = 0.34, 1.02) use was associated with greater substance use consequences. Similarly, both heavy cocaine (β = 0.84, 95% CI = 0.55, 1.14) and moderate cocaine (β = 0.33, 95% CI = 0.03, 0.63) use was associated with greater substance use consequences compared to no cocaine use. Compared to no use of the respective drug, heavy amphetamine (β = 0.85, 95% CI = 0.28, 1.42) and heavy prescription pill (β = 0.87, 95% CI = 0.46, 1.28) use was associated with greater substance use consequences. Finally, greater consequences of substance use was associated with other factors such as (1) residing in Baltimore versus Dallas (*p* <  0.001), (2) being non-Hispanic (*p* <  0.01), (3) being older (*p* <  0.001), and (4) having a higher propensity for risk taking (*p* <  0.001).

**Table 1:** Main effect model of substance use on negative substance use consequences scale (*n* = 313)

Variable	Crude STD β (95% CI)	*p*-value	Adjusted STD β (95% CI)	*p*-value
At risk of SMI	0.76 (0.55, 0.97)	< 0.001	0.59 (0.41, 0.78)	< 0.001
Binge drinking		0.17		0.80
*Heavy* versus *none*	0.15 (−0.12, 0.42)		0.06 (−0.16, 0.27)	
*Moderate* versus *none*	−0.11 (− 0.38, 0.16)		0.07 (− 0.15, 0.28)	
Opiate use		< 0.001		< 0.001
*Heavy* versus *none*	1.32 (0.99, 1.65)		0.91 (0.60, 1.22)	
*Moderate* versus *none*	0.68 (0.34, 1.02)		0.45 (0.15, 0.74)	
Marijuana use		0.13		0.02
*Heavy* versus *none*	−0.16 (−0.43, 0.10)		0.16 (− 0.07, 0.40)	
*Moderate* versus *none*	0.12 (−0.15, 0.39)		0.32 (0.09, 0.55)	
Crack/Cocaine use		< 0.001		0.01
*Heavy* versus *none*	0.84 (0.55, 1.14)		0.41 (0.16, 0.67)	
*Moderate* versus *none*	0.33 (0.03, 0.63)		0.16 (−0.08, 0.41)	
Amphetamine use		0.01		< 0.001
*Heavy* versus *none*	0.85 (0.28, 1.42)		0.96 (0.49, 1.43)	
*Moderate* versus *none*	0.29 (−0.25, 0.84)		0.43 (0.00, 0.85)	
Prescription pill use		< 0.001		0.14
*Heavy* versus *none*	0.87 (0.46, 1.28)		0.33 (−0.01, 0.66)	
*Moderate* versus *none*	0.30 (−0.12, 0.71)		−0.04 (− 0.37, 0.29)	
Age	0.22 (0.11, 0.32)	< 0.001	0.09 (−0.02, 0.20)	0.10
Male	0.02 (−0.22, 0.25)	0.90	0.22 (0.04, 0.41)	0.02
Black/African American	−0.08 (− 0.31, 0.16)	0.53	− 0.18 (0.44, 0.09)	0.20
Hispanic/Latino	−0.39 (− 0.67, − 0.12)	< 0.01	−0.12 (− 0.42, 0.17)	0.41
Risk taking score	0.33 (0.23, 0.44)	< 0.001	0.17 (0.08, 0.26)	< 0.001
Site (Dallas vs. Baltimore)	−0.54 (− 0.76, − 0.33)	< 0.001	−0.23 (− 0.43, − 0.03)	0.03

Similar, though attenuated, results were found in the adjusted models (Table 1). Adjusting for substance use, SMI risk was associated with greater substance use consequences (β = 0.59, 95% CI = 0.41, 0.78). Compared to no opiate use, both heavy opiate (β = 0.91, 95% CI = 0.60, 1.22) and moderate opiate (β = 0.45, 95% CI = 0.15, 0.74) use was associated with greater substance use consequences. Moderate marijuana use was associated with greater substance use consequences than non-use of marijuana (β = 0.32, 95% CI = 0.09, 0.55). Heavy cocaine use was associated with greater substance related consequences (β = 0.41, 95% CI = 0.16, 0.67) than no cocaine use. Finally, compared to no amphetamine use, both heavy amphetamine (β = 0.96, 95% CI = 0.49, 1.43) and moderate amphetamine (β = 0.43, 95% CI = 0.00, 0.85) use was associated greater substance use consequences.

### Differential effects of substance use by risk of SMI

Table 2 shows the interaction effects between being at risk of SMI and using a drug on substance use consequences. Binge drinking (*p* = 0.02) and opiate use (*p* = 0.02) had differential effects on substance use consequences by risk of SMI. Specifically, among those at risk of SMI, heavy binge drinking was associated with greater substance use consequences (β = 0.41, 95% CI = 0.08, 0.74), while no association between binge drinking and substance use consequences was found among those not at risk of SMI. To the contrary, opiate use had a stronger relationship with substance use consequences among those not at risk of SMI, compared to those at risk of SMI. There were no statistically significant interactions between risk of SMI and use of marijuana, crack/cocaine, prescription pills, or amphetamines on substance use consequences.

**Table 2:** Differential effects of binge drinking and opiate use by SMI status on negative consequences of use scale (*n* = 313)

Variable	At risk of SMI	Not at risk of SMI
	Crude STD β (95% CI)	Crude STD β (95% CI)
Heavy binge drinking vs. none	0.56 (0.16, 0.95)	−0.17 (− 0.48, 0.14)
Moderate binge drinking vs. none	0.05 (−0.37, 0.46)	− 0.17 (− 0.48, 0.14)
Heavy opiate use vs. none	0.71 (0.25, 1.16)	1.68 (1.27, 2.09)
Moderate opiate use vs. none	0.56 (0.08, 1.04)	0.66 (0.25, 1.07)
	Adjusted STD β (95% CI)	Adjusted STD β (95% CI)
Heavy binge drinking vs. none	0.41 (0.08, 0.74)	−0.14 (−0.40, 0.12)
Moderate binge drinking vs. none	0.13 (−0.21, 0.47)	0.06 (−0.20, 0.31)
Heavy opiate use vs. none	0.50 (0.08, 0.92)	1.27 (0.88, 1.69)
Moderate opiate use vs. none	0.37 (−0.07, 0.82)	0.53 (0.16, 0.90)

## Discussion

This study examined the relationship between substance use, risk for SMI, and negative substance use consequences among adults newly placed on probation. Contrary to previous research that utilized people who used substances but did not have SMI as their comparison group (Gonzalez et al., 2007), we found that, adjusting for type and frequency of substance use, adults at risk of SMI experienced more negative consequences than people not at risk. In fact, SMI risk was a relatively strong predictor of substance use consequences, compared to other measured variables. These findings may be explained, in part, by the supersensitivity hypothesis (Mueser et al., 1998) that suggests that people with SMI experience greater inebriation and impairment from substance use, and more consequences as a result.

Heavy alcohol use was associated with greater substance use consequences among adults at risk of a SMI, compared to those not at risk. Alcohol intoxication or withdrawal can induce psychotic, bipolar, and depressive symptoms and disorders (American Psychiatric Association, 2013). Thus, heavy alcohol use may cause more SMI symptoms that either result in more consequences or facilitate perceptions that consequences are worse (Ali et al., 2015). These findings highlight the need for the dual treatment of SMI and alcohol use. Although moderation-based approaches may be appropriate for non-alcohol dependent persons (Miller, 1983), addiction professionals are generally more cautious about proposing harm reduction strategies for clients with a history of SMI because they may encourage problematic use (Drake & Wallach, 1993). Admittedly, abstinence-based approaches may be more difficult for people who use substances to cope with symptoms of SMI. However, moderation may not be an option for people on probation because of legal requirements to abstain.

Moderate and heavy opiate use were both associated with more consequences. Interestingly, we found that heavy opiate use was associated with *fewer* consequences among those at risk of SMI, compared to those not at risk of SMI. This finding appears to contradict the hypothesized mechanisms by which substance use and SMI interact, particularly because opiate intoxication and withdrawal can cause depressive symptoms (American Psychiatric Association, 2013). However, some people who use opiates may self-select the drug because of its ability to reduce aggression (Khantzian, 1985; Mueser et al., 1998). Thus, it is possible that people at risk of SMI may be use illicit opiates to relieve short-term mental health symptoms such as interpersonal stress (e.g., feeling depressed, anxious, or guilty) and/or that the sedative effects prevent more aggressive activities (e.g., fighting with family or doing other things they regret). Thus, the interaction between substance use and mental illness on consequences may vary based on the type, amount, and combination of substances used.

A substantial proportion of the sample used prescription pills (14.7%), marijuana (63.6%), and/or stimulants, i.e., crack/cocaine (31.0%) and amphetamines (8.0%). Use of these substances did not differentially impact people at risk of a SMI. These findings may be expected for several reasons. First, there are several different types of prescription pills that are commonly abused, some of which alleviate physical or psychological ailments. As a result, the benefits and harms of prescription pills vary greatly between people. This may explain why prescription pill use was not significantly associated with negative substance use consequences in the adjusted model. Second, the use of marijuana or stimulants may have both beneficial and harmful short-term effects. Acute marijuana use can impair attention, memory, and learning (Broyd et al., 2016), but it can also alleviate stress (Crippa et al., 2009). Additionally, while stimulants can cause serious physical harm, prescription stimulant use can temporarily enhance mental and physical functioning, for instance by reducing symptoms of attention deficit hyperactivity disorder (Cramer et al., 2002) and reducing impulsivity (Pietras et al., 2003). However, there is little evidence that the consequences or protective effects of marijuana or stimulant use disproportionately affect people with SMI. The current study results should be interpreted with caution, as the entire sample was comprised of adults who reported at least some substance use.

This study did find a geographic effect related to consequences of use. Adjusting for substance use, being in Baltimore was associated with more negative substance use consequences compared to being in Dallas. There are a number of likely reasons for this: Baltimore is a denser, more urban environment, with fewer Hispanics, and higher rates of poverty, unemployment, opioid use, and crime than Dallas. Baltimore also has a larger police force per capita than Dallas. Many of these conditions are, in part, a result of the history of systematic oppression of people of color. This geographic impact suggests that some of the consequences of substance use may be accentuated by living in areas with these conditions.

Finally, we found that adults with varying recreational drug use had a differential association between SMI risk and negative substance use consequences. People who use substances are more likely to seek treatment when they perceive their substance use as causing consequences (Hajema et al., 1999; Kaskutas et al., 1997; Oser et al., 2010; Saunders et al., 2006; Weisner et al., 2002). Thus, research should continue to examine factors that affect negative consequences of substance use. In particular, future research could examine associations between specific mental health diagnoses and substance use on consequences experienced. This research will help programs become more effective at engaging different kinds of people in treatment.

### Strengths & Limitations

This study was strengthened by a relatively large, diverse sample of adults on probation in two urban areas. This study has several limitations, most notably self-report measurement of SMI risk, substance use, and related consequences. The use of convenience sampling and focus on adults on probation limit the generalizability of study findings. Being on probation may impact an individual’s perspective of consequences related to substance use, because people on probation are drug tested and there are consequences for positive drug tests. Thus, these findings may not be relevant to a general community sample of people who use substances. Moreover, people may experience legal consequences as a result of any detected substance use while on probation. Although the TLFB method has demonstrated strong reliability and validity when compared to biochemical markers in past studies (Fals-Stewart et al., 2000), associations could be influenced by recall bias.

The CODSI-SMD instrument used in this study has very high specificity (> 90%) (Sacks et al., 2007a), but like other SMI screening instruments, the instrument’s sensitivity is not as high (> 50%) (Sacks et al., 2007a). As a result, it is possible that some people who were at risk of SMI went undetected. As a result, the analyses presented here would be expected to underestimate the true relationship between SMI and substance use consequences. Preliminary evidence suggests that the CODSI-SMD is equally sensitive at detecting SMI among men and women and people of different races (Duncan et al., 2008; Sacks et al., 2007a). Moreover, studies have found that the sensitivity and specificity of the CODSI-SMD is better than other commonly used screening instruments (Sacks et al., 2007b; Sacks et al., 2007a). Because it is only three items, it is ideal for settings where researchers cannot ask an extensive battery of questions. Future studies could examine differential effects of substance use on consequences experienced between those with and without SMI using DSM criteria.

Data used in this study were cross-sectional. As a result, we are unable to determine a causal relationship between substance use, risk of mental illness, and substance use consequences. The consequences scale used in this study was positively associated with nearly every substance, suggesting that it detected consequences resulting from of a wide range of drug types. Substance use and related consequences were measured over the past 90 days, but the CODSI-SMD reflected lifetime experiences, which may raise questions about whether the experience of psychiatric symptoms are a cause of the observed differences in negative substance use consequences. However, since it is common for individuals with psychiatric symptoms such as MDD or bipolar disorder to have re-occurring mood episodes (American Psychiatric Association, 2013), it is likely that previous SMI is related to current mental health. In the current study, the CODSI-SMD clearly distinguished individuals who were likely to experience greater consequences from using substances. Our findings can help to identify the kinds of clients who may benefit from additional screening or more intensive interventions. Future research should clarify whether this observed association is due to mental health problems and/or other underlying factors.

## Conclusions

A substantial proportion of this sample (37%) of adult probationers screened at risk of having a SMI. Individuals with extensive social problems may not recognize symptoms of SMI and seek help, because their mood (e.g., depression) may seem in accordance with their life circumstances. As a result, SMI is likely to be underdiagnosed among those in the justice system. Once people with SMI are identified, clinicians should carefully consider how mental health conditions may interact with substance use to exacerbate or alleviate life problems. More research is needed to deepen our understanding of how substance use and mental health interact, and the types of treatment programs that are suitable for reducing substance use and improving mental health functionality. Additional research is needed to determine the nature of the relationship between substance use, mental health, and appropriate programming.

## Abbreviations

CJ CEST: Criminal justice client evaluation of self and treatment
CODSI-SMD: Co-occurring disorders screening instrument for severe mental disorders
DSM: The diagnostic and statistical manual of mental disorders
SIP: Short inventory of problems
SMI: Serious mental illness
SUD: Substance use disorder
TLFB: Timeline followback
